# Synthesis and crystal structure of a heterobimetallic nickel–manganese 12-metallacrown-4 methanol disolvate monohydrate compound

**DOI:** 10.1107/S205698902001316X

**Published:** 2020-10-06

**Authors:** Abigail J. Hall, Matthias Zeller, Curtis M. Zaleski

**Affiliations:** a Department of Chemistry and Biochemistry, Shippensburg University, 1871 Old Main Dr., Shippensburg, PA 17257, USA; b Department of Chemistry, Purdue University, 560 Oval Dr., West Lafayette, IN 47907, USA

**Keywords:** nickel, manganese, heterobimetallic, metallacrown, crystal structure

## Abstract

The synthesis and crystal structure of the title compound [systematic name: di-μ-acetato-tetra­kis­(μ_4_-*N*,2-dioxido­benzene-1-carboximidato)hexa­methano­ltetra­manganese(III)nickel(II) methanol disolvate monohydrate], [Mn_4_Ni(C_7_H_4_NO_3_)_4_(C_2_H_3_O_2_)_2_(CH_4_O)_6_]·2CH_4_O·H_2_O or Ni(OAc)_2_[12-MC_Mn(III)N(shi)_-4](CH_3_OH)_6_·2CH_3_OH·H_2_O, where MC is metallacrown, ^−^OAc is acetate, and shi^3−^ is salicyl­hydroximate, are reported. The macrocyclic metallacrown is positioned on an inversion center located on the Ni^II^ ion that resides in the central MC cavity. The macrocycle consists of an Mn^III^–N–O repeat unit that recurs four times to generate an overall square-shaped mol­ecule. Both the Ni^II^ and Mn^III^ ions are six-coordinate with an octa­hedral geometry. In addition, the Mn^III^ ions possess an elongated Jahn–Teller distortion along the *z*-axis of the coordination environment. The inter­stitial water mol­ecule is slightly offset from and disordered about an inversion center.

## Abstract 2

The metallacrown compound, Ni^II^(OAc)_2_[12-MC_Mn(III)N(shi)_-4](CH_3_OH)_6_·2CH_3_OH·H_2_O, where MC is metallacrown, shi^3−^ is salicyl­hydroximate, and ^−^OAc is acetate, has an overall square shape that captures an Ni^II^ ion in a central cavity while the ring of the metallamacrocycle contains four Mn^III^ ions. Two acetate anions, which are located on opposite faces of the MC, form bridges between the central Ni^II^ ion and two of the ring Mn^III^ ions.

## Chemical context   

Since their recognition in 1989 by Pecoraro, metallacrowns (MC) have proven to be a versatile class of metallamacrocycles with applications such as single-mol­ecule magnets, magnetorefrigerants and optical imaging agents (Mezei *et al.*, 2007 ▸; Nguyen & Pecoraro, 2017 ▸; Lutter *et al.*, 2018 ▸). The archetypal metallacrown consists of a cyclic metal–nitro­gen–oxygen repeat unit that generates a central cavity that is capable of binding a metal ion. Initially, homometallic compounds were produced; however, heterobimetallic systems were soon generated that typically contained trans­ition-metal ions in the ring metal position and either alkali or lanthanide ions captured in the central cavity of the MC (Pecoraro *et al.*, 1997 ▸; Mezei *et al.*, 2007 ▸). In addition, heterotrimetallic systems that bind both alkali and lanthanide ions have been reported since 2014 (Azar *et al.*, 2014 ▸). One area lacking is the use of two different transition-metal ions in an archetypal MC. While several examples of heterobimetallic 3*d* ‘collapsed’ metallacrowns, species without a central MC cavity and thus no central metal ion (Psomas *et al.*, 2001 ▸; Gole *et al.*, 2010 ▸), and inverse metallacrowns, species that bind a non-metal atom in the central MC cavity to the ring metal ions (Szyrwiel *et al.*, 2013 ▸; Shiga *et al.*, 2014 ▸; Zhang *et al.*, 2014 ▸; Nesterova *et al.*, 2015 ▸), have been reported, only two heterobimetallic 3*d* archetypal 12-MC-4 compounds have been described to date. In 2014, Happ and Rentschler reported a Cu^II^(DMF)_2_Cl_2_[12-MC_Fe(III)N(shi)_-4](DMF)_4_·2DMF compound that contains Fe^III^ ions in the ring positions and a Cu^II^ ion captured in the central MC cavity (Happ & Rentschler, 2014 ▸). Recently we described the structure of (TMA)_2_{Mn(OAc)_2_[12-MC_Mn(III)Cu(II)N(shi)_-4](CH_3_OH)}·2.90CH_3_OH that consists of alternating Cu^II^ and Mn^III^ ions about the MC ring and an Mn^II^ ion bound to the central MC cavity (Lewis *et al.*, 2020 ▸). Herein we report a third heterobimetallic 3*d* archetypal 12-MC-4 compound: Ni^II^(OAc)_2_[12-MC_Mn(III)N(shi)_-4](CH_3_OH)_6_·2CH_3_OH·H_2_O, **1**, that contains ring Mn^III^ ions and a Ni^II^ ion captured in the central MC cavity. Future work will focus on the magnetic properties of the compound as the similar Mn(OAc)_2_[12-MC_Mn(III)N(shi)_-4] (Zaleski *et al.*, 2011 ▸) and {Mn(OAc)_2_[12-MC_Mn(III)Cu(II)N(shi)_-4]}^2−^ (Lewis *et al.*, 2020 ▸) systems behave as single-mol­ecule magnets.
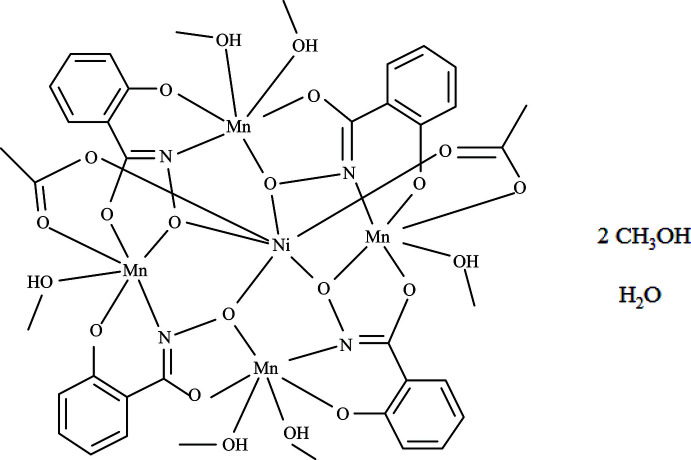


## Structural commentary   

**Figure 1:**
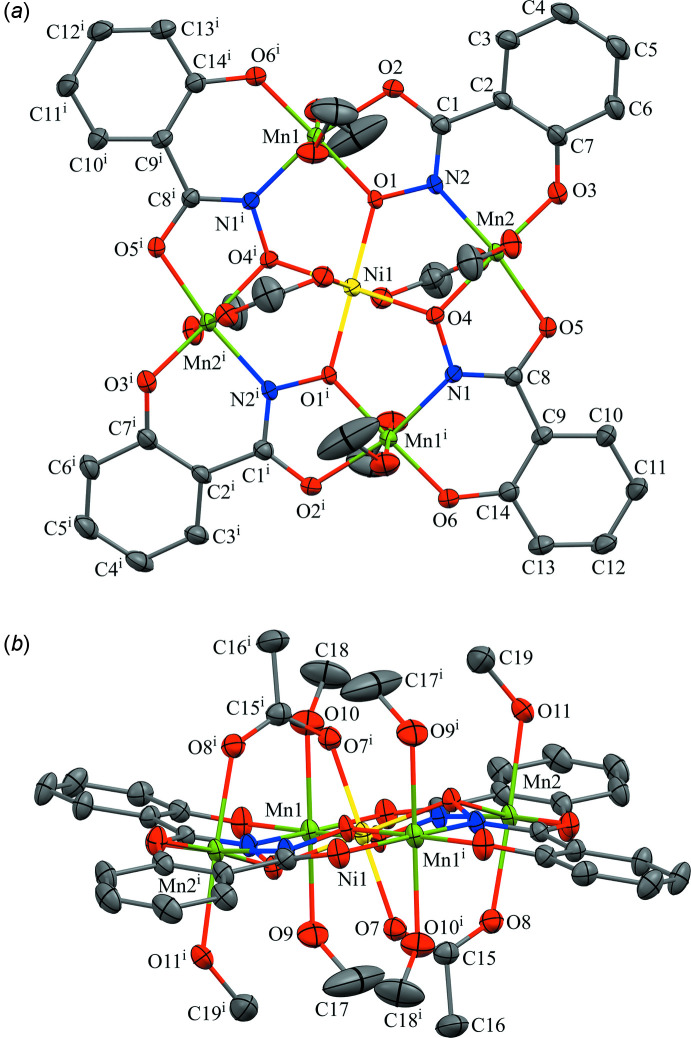
The mol­ecular structure of the title compound, (*a*) top view with only the metal atoms and shi^3−^ ligands labeled for clarity and (*b*) side view with only the metal atoms and axial ligands labeled for clarity. The displacement ellipsoids are at the 50% probability level. For clarity, hydrogen atoms and solvent mol­ecules have been omitted. [Color scheme: yellow–Ni^II^, green–Mn^III^, red–O, blue–N and gray–C; symmetry code: (i) −*x* + 1, −*y* + 1, −*z* + 1.]

The title metallacrown compound is positioned about an inversion center located on the Ni^II^ ion that resides in the central MC cavity (Fig. 1 ▸). The metallacrown macrocycle possesses an Mn^III^–N–O repeat unit that generates an approximately square mol­ecule due to the fused five- and six-membered chelate rings of the shi^3−^ ligand that place the Mn^III^ ions 90^o^ relative to each other. The oxime oxygen atoms of the shi^3−^ ligands generate the MC cavity and also bind the central Ni^II^ ion. Two acetate anions, which bind on opposite faces of the MC, tether the Ni^II^ ion to the MC by forming three atom bridges to two of the ring Mn^III^ ions. In addition to average bond lengths and bond-valence-sum (BVS) values (Table 1 ▸; Liu & Thorp, 1993 ▸), the oxidation state assignments of the Ni^II^ and Mn^III^ ions are supported by overall mol­ecular charges, where one Ni^II^ and four Mn^III^ ions are counterbalanced by four shi^3−^ and two acetate anions.

The central Ni^II^ ion is six-coordinate with an octa­hedral geometry as verified by a SHAPE analysis (*SHAPE 2.1*; Llunell *et al.*, 2013 ▸; Pinsky & Avnir, 1998 ▸). Continuous shape measure (CShM) values of less than 1.0 indicate only minor distortions from the ideal geometry (Cirera *et al.*, 2005 ▸); thus, the CShM value of 0.164 for the octa­hedral geometry clearly defines the shape about the Ni^II^ ion (Table 2 ▸). The coordination environment is comprised of four oxime oxygen from four shi^3−^ ligands in the equatorial plane and two axial carboxyl­ate oxygen atoms of two acetate anions. As mentioned above, the acetate anions bind on opposite faces of the MC and connect the Ni^II^ ion to two Mn^III^ ions (Mn2). The acetate binding motif is different than the analogous homometallic Mn^II^(OAc)_2_[12-MC_Mn(III)N(shi)_-4](DMF)_6_·2DMF, where the acetate anions bind on the same face of the MC and the central Mn^II^ ion exhibits a geometry that is best described as a trigonal prism (Lah & Pecoraro, 1989 ▸).

The ring Mn^III^ ions (Mn1 and Mn2) are six-coordinate with a tetra­gonally distorted octa­hedral geometry (Table 2 ▸). The Jahn–Teller elongation, typical of a high-spin 3*d* ^4^ ion, is located along the *z*-axis of each Mn^III^ ion. For both Mn1 and Mn2, the equatorial coordination environment is composed of *trans* chelate rings from two shi^3−^ ligands. A five-membered chelate ring is generated from an oxime oxygen atom and a carbonyl oxygen atom of one shi^3−^ ligand, and a six-membered chelate ring is produced by an oxime nitro­gen atom and a phenolate oxygen atom of the second shi^3−^ ligand. For Mn1 the axial atoms are oxygen atoms from two methanol mol­ecules, while for Mn2 the axial atoms are an oxygen atom from a methanol mol­ecule and a carboxyl­ate oxygen atom from an acetate anion.

In addition, solvent methanol and water mol­ecules are located in the structure, and the methanol mol­ecules form hydrogen bonds to the metallacrown. The water mol­ecule associated with O13 is slightly offset from and disordered about an inversion center.

## Supra­molecular features   

**Figure 2:**
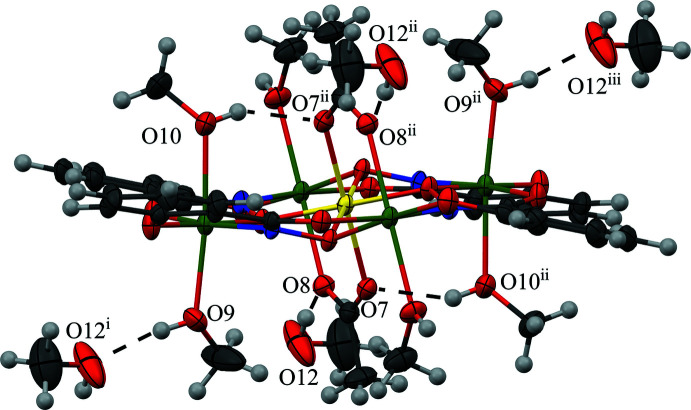
Intra­molecular hydrogen bonding (dashed lines) between the coordinated methanol mol­ecule (O10) and a carboxyl­ate oxygen atom (O7) of the bridging acetate anion, and inter­molecular hydrogen bonding (dashed lines) of the inter­stitial methanol mol­ecule (O12) with a coordinated methanol mol­ecule (O9) and a carboxyl­ate oxygen atom (O8) of the bridging acetate anion. [Symmetry codes: (i) −*x* + 2, −*y* + 1, −*z* + 1; (ii) −*x* + 1, −*y* + 1, −*z* + 1; (iii) *x* − 1, *y*, *z*.]

**Figure 3:**
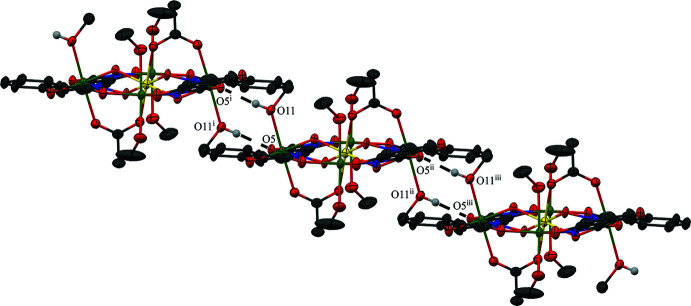
Inter­molecular hydrogen bonding (dashed lines) between the carboxyl­ate oxygen atom (O5) of a shi^3−^ ligand and a coordinated methanol mol­ecule (O11) of a neighboring metallacrown that then generates a one-dimensional chain. For clarity only the hydrogen atoms involved in the inter­actions have been included. [Symmetry codes: (i) −*x* + 1, −*y* + 2, −*z* + 1; (ii) −*x* + 1, −*y* + 1, −*z* + 1; (iii) *x*, *y* − 1, *z*.]

The coordinated and inter­stitial methanol mol­ecules of **1** participate in several hydrogen bonds (Figs. 2 ▸ and 3 ▸, Table 3 ▸). The hydroxyl group of the methanol mol­ecule associated with O9 and coordinated to Mn1 forms a hydrogen bond to an oxygen atom (O12) of an inter­stitial methanol mol­ecule. In addition, the hy­droxy group of another methanol mol­ecule associated with O10 and coordinated to Mn1 forms an intra­molecular hydrogen bond to a carboxyl­ate oxygen atom (O7) of an acetate anion. Also the hydroxyl group of the inter­stitial methanol mol­ecule associated with O12 forms a hydrogen bond to the other carboxyl­ate oxygen atom (O8) of the acetate anion. Lastly, a one-dimensional chain of metallacrowns is mediated by the hydroxyl group of a methanol mol­ecule associated with O11 and coordinated to Mn2 that forms a hydrogen bond to a carboxyl­ate oxygen atom (O5) of a shi^3−^ ligand of a neighboring metallacrown. As a symmetry-equivalent hydrogen bond also occurs on the opposite side of the MC, a one-dimensional chain is established (Fig. 3 ▸). The connection between the neighboring MCs, the hydrogen bonds between the MC and the inter­stitial methanol mol­ecules, and pure van der Waals forces contribute to the overall packing of **1**.

## Database survey   

A survey of the Cambridge Structural Database (CSD version 5.41, update May 2020, Groom *et al.*, 2016 ▸) reveals that there are three other heterobimetallic manganese-nickel MC compounds. The first reported manganese-nickel MC is a ‘collapsed’ metallacrown as it does not contain a central cavity. The structure has an *M*–N–O repeat unit but two of the oxime oxygen atoms bind to ring metal ions across the potential central cavity, thus collapsing the cavity and preventing the binding of a central metal ion. The compound [12-MC_Ni(II)Mn(III)N(shi)2(pko)2_-4](OAc)_2_ (QOCXAH; Psomas *et al.*, 2001 ▸), where pko^−^ is 2,2′-di­pyridyl­ketonoximate, contains both Mn^III^ and Ni^II^ ions in the MC ring positions with the metals arranged in an alternating pattern. The two other compounds can both be considered dimers of inverse 9-MC-3 systems, where each MC binds a μ_3_-O in the central cavity instead of a metal ion. In both compounds, two inverse 9-MC-3 units, each based on an Mn^III^_2_Ni^II^ core, are linked together to form a dimer. The main difference between the structures is the MC framework ligand: salicylaldoxime (XIFGUQ; Szyrwiel *et al.*, 2013 ▸) or 5-chloro­salicyl­aldehyde oxime (LOKHIE; Zhang *et al.*, 2014 ▸). Thus, **1** represents the only manganese–nickel archetypal MC structure type as **1** contains a central metal ion.

## Synthesis and crystallization   

Manganese(II) acetate tetra­hydrate (99+%), tetra­ethyl­ammonium acetate tetra­hydrate (99%), salicyl­hydroxamic acid (99%), nickel(II) acetate tetra­hydrate (99.995%), *N*,*N*-di­methyl­formamide (DMF, Certified ACS grade) and methanol (ACS grade) were purchased from Acros Organics, Acros Organics, Alfa Aesar, Sigma–Aldrich, BDH Chemicals and Pharmco-AAPER, respectively. All reagents were used as received without further purification.

Tetra­ethyl­ammonium acetate tetra­hydrate (4 mmol, 1.0462 g) and salicyl­hydroxamic acid (2 mmol, 0.3063 g) were dissolved in 4 mL of DMF and 4 mL of methanol, resulting in a clear orange solution. In two separate vessels, nickel(II) acetate tetra­hydrate (0.125 mmol, 0.0312 g) was dissolved in 4 mL of DMF and 4 mL of methanol resulting in a green–blue solution and manganese(II) acetate tetra­hydrate (2 mmol, 0.4909 g) was dissolved in 4 mL of DMF and 4 mL of methanol resulting in an clear orange solution. The manganese(II) acetate solution was then added to the tetra­ethyl­ammonium acetate/salicyl­hydroxamic acid solution resulting in a brown solution. The nickel(II) acetate was then immediately added and no color change was detected; however, the formation of a precipitate was observed. The mixture was then left to stir overnight and subsequently gravity filtered the next day. The filtrate was a dark orange–brown solution and no precipitate was recovered. Slow evaporation of the filtrate at room temperature afforded X-ray quality dark-brown block-shaped crystals after 16 weeks. A small fraction of crystals and mother liquor were separated for analysis by single crystal X-ray diffraction. The remaining crystals were washed with cold DMF and dried. The percent yield was 22% based on nickel(II) acetate tetra­hydrate.

## Refinement   

Crystal data, data collection and structure refinement details are summarized in Table 4 ▸. An electron density region disordered around an inversion center was refined as a water mol­ecule located slightly offset of the inversion center. The water hydrogen-atom positions were initially refined and O—H and H⋯H distances were restrained to 0.84 (2) and 1.36 (2) Å, respectively, and further restrained based on hydrogen-bonding considerations while a damping factor was applied. In the final refinement cycles the hydrogen atoms were constrained to ride on the oxygen carrier atom. The displacement parameters of the water O atom were restrained to be close to isotropic. For the methanol mol­ecules, the O—H bond distance was also restrained to 0.84 (2) Å. The *U*_iso_ values for the O—H hydrogen atoms (water and methanol) were set to a multiple of the value of the carrying oxygen atom (1.5 times). All other hydrogen atoms were placed in calculated positions and refined as riding on their carrier atoms with C—H distances of 0.95 Å for *sp* ^2^ carbon atoms and 0.98 Å for methyl carbon atoms. The *U*_iso_ values for hydrogen atoms were set to a multiple of the value of the carrying carbon atom (1.2 times for *sp* ^2^-hybridized carbon atoms or 1.5 times for methyl carbon atoms).

## Supplementary Material

Crystal structure: contains datablock(s) I, global. DOI: 10.1107/S205698902001316X/is5558sup1.cif

Structure factors: contains datablock(s) I. DOI: 10.1107/S205698902001316X/is5558Isup2.hkl

CCDC reference: 2034558

Additional supporting information:  crystallographic information; 3D view; checkCIF report

## Figures and Tables

**Table 1:** Average bond length (Å) and bond-valence-sum (BVS) values (v.u.) used to support assigned oxidation states of the nickel and manganese ions of **1**

	Avg. bond length	BVS value	Assigned oxidation state
Ni1	2.021	2.34	2+
Mn1	2.031	3.06	3+
Mn2	2.026	3.06	3+

**Table 2:** Continuous Shapes Measures (CShM) values for the geometry about the six-coordinate central Ni^II^ and ring Mn^III^ ions of **1**

Shape	Hexagon (*D*_6h_)	Penta­gonal pyramid (*C*_5v_)	Octa­hedron (*O*_h_)	Trigonal prism (*D*_3h_)	Johnson penta­gonal pyramid (J2; *C*_5v_)
Ni1	31.656	29.242	0.164	16.201	32.416
Mn1	31.782	26.077	1.106	13.698	29.622
Mn2	31.099	26.575	0.821	15.377	29.523

**Table 3:** Hydrogen-bond geometry (Å, °)

*D*—H⋯*A*	*D*—H	H⋯*A*	*D*⋯*A*	*D*—H⋯*A*
O9—H9*O*⋯O12^i^	0.82 (2)	1.82 (2)	2.630 (4)	172 (4)
O10—H10*O*⋯O7^ii^	0.84 (2)	1.97 (3)	2.713 (3)	148 (4)
O11—H11*O*⋯O5^iii^	0.83 (2)	1.95 (2)	2.778 (2)	178 (4)
O12—H12*A*⋯O8	0.84	1.94	2.743 (3)	159

Symmetry codes: (i) 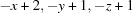; (ii) 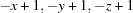; (iii) 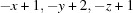.

**Table 4:** Experimental details

Crystal data	
Chemical formula	[Mn_4_Ni(C_7_H_4_NO_3_)_4_(C_2_H_3_O_2_)_2_(CH_4_O)_6_]·2CH_4_O·H_2_O
*M*_r_	1271.35
Crystal system, space group	Triclinic, *P* 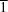
Temperature (K)	150
*a*, *b*, *c* (Å)	10.3647 (7), 10.7781 (8), 11.8303 (8)
α, β, γ (°)	85.318 (3), 86.231 (3), 77.583 (3)
*V* (Å^3^)	1284.78 (16)
*Z*	1
Radiation type	Mo *K*α
μ (mm^−1^)	1.40
Crystal size (mm)	0.24 × 0.22 × 0.13

Data collection	
Diffractometer	Bruker AXS D8 Quest CMOS diffractometer
Absorption correction	Multi-scan (*SADABS2016/2*; Krause *et al.*, 2015 ▸)
*T*_min_, *T*_max_	0.616, 0.746
No. of measured, independent and observed [*I* > 2σ(*I*)] reflections	13573, 7767, 6204
*R*_int_	0.026
(sin θ/λ)_max_ (Å^−1^)	0.717

Refinement	
*R*[*F* ^2^ > 2σ(*F* ^2^)], *wR*(*F* ^2^), *S*	0.050, 0.137, 1.07
No. of reflections	7767
No. of parameters	355
No. of restraints	9
H-atom treatment	H atoms treated by a mixture of independent and constrained refinement
Δρ_max_, Δρ_min_ (e Å^−3^)	1.79, −1.02

Computer programs: *APEX3* and *SAINT* (Bruker, 2018 ▸), *SHELXS97* (Sheldrick, 2008 ▸), *SHELXL2018/3* (Sheldrick, 2015 ▸), *SHELXLE* (Hübschle *et al.*, 2011 ▸), *Mercury* (Macrae *et al.*, 2020 ▸) and *publCIF* (Westrip, 2010 ▸).

## supplementary crystallographic information

### Crystal data

**Table:**

[Mn_4_Ni(C_7_H_4_NO_3_)_4_(C_2_H_3_O_2_)_2_(CH_4_O)_6_]·2CH_4_O·H_2_O	*Z* = 1
*M**_r_* = 1271.35	*F*(000) = 652
Triclinic, *P*1	*D*_x_ = 1.643 Mg m^−^^3^
*a* = 10.3647 (7) Å	Mo *K*α radiation, λ = 0.71073 Å
*b* = 10.7781 (8) Å	Cell parameters from 9780 reflections
*c* = 11.8303 (8) Å	θ = 2.6–30.6°
α = 85.318 (3)°	µ = 1.40 mm^−^^1^
β = 86.231 (3)°	*T* = 150 K
γ = 77.583 (3)°	Block, brown
*V* = 1284.78 (16) Å^3^	0.24 × 0.22 × 0.13 mm

### Data collection

**Table:**

Bruker AXS D8 Quest CMOS diffractometer	7767 independent reflections
Radiation source: fine focus sealed tube X-ray source	6204 reflections with *I* > 2σ(*I*)
Triumph curved graphite crystal monochromator	*R*_int_ = 0.026
Detector resolution: 10.4167 pixels mm^-1^	θ_max_ = 30.6°, θ_min_ = 2.5°
ω and phi scans	*h* = −14→14
Absorption correction: multi-scan (*SADABS2016/2*; Krause *et al.*, 2015)	*k* = −15→15
*T*_min_ = 0.616, *T*_max_ = 0.746	*l* = −16→16
13573 measured reflections	

### Refinement

**Table:**

Refinement on *F*^2^	Primary atom site location: structure-invariant direct methods
Least-squares matrix: full	Secondary atom site location: difference Fourier map
*R*[*F*^2^ > 2σ(*F*^2^)] = 0.050	Hydrogen site location: mixed
*wR*(*F*^2^) = 0.137	H atoms treated by a mixture of independent and constrained refinement
*S* = 1.07	*w* = 1/[σ^2^(*F*_o_^2^) + (0.072*P*)^2^ + 1.2905*P*] where *P* = (*F*_o_^2^ + 2*F*_c_^2^)/3
7767 reflections	(Δ/σ)_max_ = 0.030
355 parameters	Δρ_max_ = 1.79 e Å^−^^3^
9 restraints	Δρ_min_ = −1.02 e Å^−^^3^

### Special details

**Table:**

Geometry. All esds (except the esd in the dihedral angle between two l.s. planes) are estimated using the full covariance matrix. The cell esds are taken into account individually in the estimation of esds in distances, angles and torsion angles; correlations between esds in cell parameters are only used when they are defined by crystal symmetry. An approximate (isotropic) treatment of cell esds is used for estimating esds involving l.s. planes.
Refinement. A single electron density disordered around an inversion center was refined as a water molecule located slightly offset of the inversion center. The water H atom positions were initially refined and O-H and H···H distances were restrained to 0.84 (2) and 1.36 (2) Angstrom, respectively and further restrained based on hydrogen bonding considerations while a damping factor was applied. In the final refinement cycles the H atoms were constrained to ride on the oxygen carrier atom.

### Fractional atomic coordinates and isotropic or equivalent isotropic displacement parameters (Å^2^)

**Table:**

	*x*	*y*	*z*	*U*_iso_*/*U*_eq_	Occ. (<1)
Ni1	0.5000	0.5000	0.5000	0.01808 (10)	
Mn1	0.72582 (4)	0.43090 (3)	0.28526 (3)	0.01909 (10)	
Mn2	0.59489 (3)	0.76188 (3)	0.52263 (3)	0.01796 (9)	
O1	0.65814 (17)	0.52833 (15)	0.40892 (14)	0.0203 (3)	
O2	0.81297 (18)	0.57917 (17)	0.25092 (15)	0.0256 (4)	
O3	0.72951 (19)	0.85163 (17)	0.49693 (15)	0.0272 (4)	
O4	0.44721 (17)	0.67981 (15)	0.54628 (13)	0.0191 (3)	
O5	0.50532 (17)	0.84616 (15)	0.65760 (14)	0.0209 (3)	
O6	0.21353 (19)	0.66578 (18)	0.83693 (15)	0.0278 (4)	
O7	0.61523 (18)	0.44755 (16)	0.64128 (14)	0.0239 (3)	
O8	0.70764 (18)	0.61703 (17)	0.64174 (15)	0.0259 (4)	
O9	0.8980 (2)	0.3245 (2)	0.37362 (19)	0.0349 (4)	
H9O	0.964 (3)	0.307 (4)	0.331 (3)	0.052*	
O10	0.5606 (2)	0.5444 (2)	0.17778 (16)	0.0344 (4)	
H10O	0.498 (3)	0.576 (4)	0.222 (3)	0.052*	
O11	0.4808 (2)	0.90151 (17)	0.39868 (17)	0.0300 (4)	
H11O	0.486 (4)	0.977 (2)	0.384 (3)	0.045*	
O12	0.8752 (3)	0.7344 (5)	0.7432 (3)	0.0847 (13)	
H12A	0.8150	0.7177	0.7066	0.127*	
O13	1.015 (3)	0.4661 (18)	−0.003 (2)	0.156 (6)	0.5
H13A	0.9779	0.5158	0.0521	0.233*	0.5
H13B	0.9842	0.3968	0.0223	0.233*	0.5
N1	0.3884 (2)	0.69063 (18)	0.65720 (15)	0.0182 (4)	
N2	0.6789 (2)	0.65323 (18)	0.39992 (16)	0.0199 (4)	
C1	0.7675 (2)	0.6685 (2)	0.31880 (19)	0.0213 (4)	
C2	0.8140 (2)	0.7887 (2)	0.3086 (2)	0.0220 (4)	
C3	0.8848 (3)	0.8180 (2)	0.2086 (2)	0.0273 (5)	
H3	0.8979	0.7621	0.1488	0.033*	
C4	0.9360 (3)	0.9269 (3)	0.1955 (3)	0.0338 (6)	
H4	0.9836	0.9460	0.1272	0.041*	
C5	0.9169 (3)	1.0079 (3)	0.2831 (3)	0.0342 (6)	
H5	0.9525	1.0825	0.2747	0.041*	
C6	0.8469 (3)	0.9817 (2)	0.3826 (2)	0.0301 (5)	
H6	0.8353	1.0383	0.4417	0.036*	
C7	0.7929 (2)	0.8724 (2)	0.3972 (2)	0.0232 (5)	
C8	0.4211 (2)	0.7826 (2)	0.70775 (18)	0.0183 (4)	
C9	0.3615 (2)	0.8147 (2)	0.82130 (18)	0.0203 (4)	
C10	0.4022 (3)	0.9118 (3)	0.8729 (2)	0.0268 (5)	
H10	0.4670	0.9525	0.8345	0.032*	
C11	0.3501 (3)	0.9492 (3)	0.9781 (2)	0.0327 (6)	
H11	0.3787	1.0151	1.0116	0.039*	
C12	0.2558 (3)	0.8900 (3)	1.0346 (2)	0.0316 (6)	
H12	0.2203	0.9151	1.1073	0.038*	
C13	0.2134 (3)	0.7951 (3)	0.9856 (2)	0.0259 (5)	
H13	0.1483	0.7557	1.0252	0.031*	
C14	0.2643 (2)	0.7553 (2)	0.87871 (19)	0.0216 (4)	
C15	0.6980 (3)	0.5059 (2)	0.6754 (2)	0.0258 (5)	
C16	0.7891 (4)	0.4355 (3)	0.7642 (3)	0.0443 (8)	
H16A	0.8066	0.3439	0.7540	0.066*	
H16B	0.7475	0.4518	0.8399	0.066*	
H16C	0.8726	0.4649	0.7565	0.066*	
C17	0.9312 (5)	0.3374 (7)	0.4821 (4)	0.107 (3)	
H17A	1.0278	0.3194	0.4855	0.129*	
H17B	0.8951	0.2773	0.5352	0.129*	
H17C	0.8945	0.4245	0.5030	0.129*	
C18	0.5752 (4)	0.6311 (4)	0.0836 (3)	0.0627 (12)	
H18A	0.6480	0.5919	0.0324	0.094*	
H18B	0.5944	0.7086	0.1103	0.094*	
H18C	0.4930	0.6529	0.0430	0.094*	
C19	0.3701 (4)	0.8873 (3)	0.3386 (3)	0.0447 (8)	
H19A	0.3931	0.8898	0.2569	0.067*	
H19B	0.2946	0.9568	0.3545	0.067*	
H19C	0.3468	0.8055	0.3633	0.067*	
C20	0.8243 (6)	0.7872 (6)	0.8340 (5)	0.0872 (18)	
H20A	0.7712	0.8719	0.8138	0.105*	
H20B	0.8951	0.7951	0.8822	0.105*	
H20C	0.7679	0.7347	0.8754	0.105*	

### Atomic displacement parameters (Å^2^)

**Table:**

	*U*^11^	*U*^22^	*U*^33^	*U*^12^	*U*^13^	*U*^23^
Ni1	0.0219 (2)	0.01533 (19)	0.01792 (18)	−0.00658 (15)	0.00285 (14)	−0.00226 (14)
Mn1	0.02230 (19)	0.01651 (17)	0.01929 (16)	−0.00688 (13)	0.00547 (12)	−0.00386 (12)
Mn2	0.02245 (18)	0.01363 (16)	0.01896 (16)	−0.00712 (13)	0.00365 (12)	−0.00290 (12)
O1	0.0270 (8)	0.0128 (7)	0.0225 (7)	−0.0092 (6)	0.0070 (6)	−0.0038 (6)
O2	0.0284 (9)	0.0234 (8)	0.0260 (8)	−0.0104 (7)	0.0107 (7)	−0.0053 (7)
O3	0.0324 (10)	0.0250 (8)	0.0282 (8)	−0.0149 (7)	0.0028 (7)	−0.0055 (7)
O4	0.0251 (8)	0.0171 (7)	0.0159 (7)	−0.0070 (6)	0.0043 (6)	−0.0030 (6)
O5	0.0260 (9)	0.0164 (7)	0.0218 (7)	−0.0078 (6)	0.0024 (6)	−0.0045 (6)
O6	0.0324 (10)	0.0275 (9)	0.0264 (8)	−0.0139 (8)	0.0112 (7)	−0.0094 (7)
O7	0.0271 (9)	0.0227 (8)	0.0223 (8)	−0.0066 (7)	0.0000 (7)	−0.0004 (6)
O8	0.0261 (9)	0.0262 (9)	0.0265 (8)	−0.0084 (7)	−0.0030 (7)	0.0003 (7)
O9	0.0228 (10)	0.0386 (11)	0.0400 (11)	0.0006 (8)	0.0007 (8)	−0.0037 (9)
O10	0.0312 (11)	0.0416 (11)	0.0267 (9)	−0.0048 (9)	0.0037 (8)	0.0075 (8)
O11	0.0385 (11)	0.0183 (8)	0.0342 (9)	−0.0087 (7)	−0.0071 (8)	0.0038 (7)
O12	0.0352 (14)	0.156 (4)	0.076 (2)	−0.0347 (19)	0.0118 (14)	−0.064 (2)
O13	0.170 (9)	0.188 (12)	0.099 (5)	−0.036 (9)	0.009 (6)	0.026 (9)
N1	0.0229 (9)	0.0162 (8)	0.0158 (8)	−0.0051 (7)	0.0033 (7)	−0.0037 (6)
N2	0.0247 (10)	0.0144 (8)	0.0224 (8)	−0.0090 (7)	0.0039 (7)	−0.0026 (7)
C1	0.0225 (11)	0.0198 (10)	0.0216 (10)	−0.0061 (8)	0.0018 (8)	0.0010 (8)
C2	0.0194 (11)	0.0181 (10)	0.0292 (11)	−0.0081 (8)	0.0040 (9)	0.0018 (9)
C3	0.0238 (12)	0.0255 (11)	0.0316 (12)	−0.0070 (9)	0.0073 (9)	0.0009 (10)
C4	0.0276 (13)	0.0286 (13)	0.0435 (15)	−0.0095 (11)	0.0084 (11)	0.0089 (11)
C5	0.0299 (14)	0.0229 (12)	0.0506 (16)	−0.0116 (10)	0.0051 (12)	0.0034 (11)
C6	0.0309 (14)	0.0200 (11)	0.0418 (14)	−0.0117 (10)	0.0027 (11)	−0.0021 (10)
C7	0.0179 (11)	0.0193 (10)	0.0326 (12)	−0.0053 (8)	0.0003 (9)	0.0001 (9)
C8	0.0186 (10)	0.0170 (9)	0.0188 (9)	−0.0031 (8)	0.0005 (8)	−0.0019 (8)
C9	0.0216 (11)	0.0224 (10)	0.0164 (9)	−0.0032 (8)	0.0014 (8)	−0.0043 (8)
C10	0.0273 (12)	0.0303 (12)	0.0251 (11)	−0.0091 (10)	0.0031 (9)	−0.0110 (10)
C11	0.0294 (13)	0.0437 (15)	0.0297 (12)	−0.0135 (12)	0.0040 (10)	−0.0200 (11)
C12	0.0279 (13)	0.0466 (16)	0.0219 (11)	−0.0090 (12)	0.0045 (9)	−0.0132 (11)
C13	0.0253 (12)	0.0313 (12)	0.0197 (10)	−0.0039 (10)	0.0035 (9)	−0.0031 (9)
C14	0.0217 (11)	0.0211 (10)	0.0212 (10)	−0.0033 (8)	0.0010 (8)	−0.0018 (8)
C15	0.0257 (12)	0.0288 (12)	0.0213 (10)	−0.0024 (10)	−0.0002 (9)	−0.0020 (9)
C16	0.0485 (19)	0.0448 (17)	0.0405 (16)	−0.0108 (14)	−0.0223 (14)	0.0105 (14)
C17	0.070 (3)	0.176 (6)	0.044 (2)	0.061 (4)	−0.022 (2)	−0.035 (3)
C18	0.051 (2)	0.073 (3)	0.051 (2)	−0.0006 (19)	0.0063 (17)	0.033 (2)
C19	0.058 (2)	0.0368 (16)	0.0437 (17)	−0.0176 (15)	−0.0181 (15)	0.0064 (13)
C20	0.092 (4)	0.097 (4)	0.088 (4)	−0.048 (3)	0.031 (3)	−0.047 (3)

### Geometric parameters (Å, º)

**Table:**

Ni1—O1	1.9673 (16)	N2—C1	1.309 (3)
Ni1—O1^i^	1.9673 (16)	C1—C2	1.470 (3)
Ni1—O4^i^	2.0082 (15)	C2—C3	1.401 (3)
Ni1—O4	2.0082 (15)	C2—C7	1.414 (4)
Ni1—O7	2.0874 (17)	C3—C4	1.382 (4)
Ni1—O7^i^	2.0874 (17)	C3—H3	0.9500
Mn1—O6^i^	1.8480 (17)	C4—C5	1.387 (4)
Mn1—O1	1.8799 (16)	C4—H4	0.9500
Mn1—N1^i^	1.9990 (19)	C5—C6	1.383 (4)
Mn1—O2	2.0003 (18)	C5—H5	0.9500
Mn1—O9	2.182 (2)	C6—C7	1.404 (3)
Mn1—O10	2.275 (2)	C6—H6	0.9500
Mn2—O3	1.8578 (18)	C8—C9	1.477 (3)
Mn2—O4	1.9208 (17)	C9—C10	1.405 (3)
Mn2—N2	1.9772 (19)	C9—C14	1.415 (3)
Mn2—O5	1.9789 (17)	C10—C11	1.380 (3)
Mn2—O8	2.2048 (18)	C10—H10	0.9500
Mn2—O11	2.2170 (18)	C11—C12	1.386 (4)
O1—N2	1.403 (2)	C11—H11	0.9500
O2—C1	1.294 (3)	C12—C13	1.378 (4)
O3—C7	1.339 (3)	C12—H12	0.9500
O4—N1	1.413 (2)	C13—C14	1.402 (3)
O5—C8	1.306 (3)	C13—H13	0.9500
O6—C14	1.335 (3)	C15—C16	1.504 (4)
O6—Mn1^i^	1.8480 (17)	C16—H16A	0.9800
O7—C15	1.270 (3)	C16—H16B	0.9800
O8—C15	1.254 (3)	C16—H16C	0.9800
O9—C17	1.377 (5)	C17—H17A	0.9800
O9—H9O	0.820 (19)	C17—H17B	0.9800
O10—C18	1.416 (4)	C17—H17C	0.9800
O10—H10O	0.838 (18)	C18—H18A	0.9800
O11—C19	1.431 (4)	C18—H18B	0.9800
O11—H11O	0.834 (18)	C18—H18C	0.9800
O12—C20	1.285 (5)	C19—H19A	0.9800
O12—H12A	0.8400	C19—H19B	0.9800
O13—H13A	0.8912	C19—H19C	0.9800
O13—H13B	0.8940	C20—H20A	0.9800
N1—C8	1.313 (3)	C20—H20B	0.9800
N1—Mn1^i^	1.9991 (19)	C20—H20C	0.9800
			
O1—Ni1—O1^i^	180.0	O2—C1—C2	121.6 (2)
O1—Ni1—O4^i^	85.54 (6)	N2—C1—C2	118.0 (2)
O1^i^—Ni1—O4^i^	94.46 (6)	C3—C2—C7	119.7 (2)
O1—Ni1—O4	94.46 (6)	C3—C2—C1	118.1 (2)
O1^i^—Ni1—O4	85.54 (6)	C7—C2—C1	122.2 (2)
O4^i^—Ni1—O4	180.0	C4—C3—C2	121.0 (3)
O1—Ni1—O7	89.30 (7)	C4—C3—H3	119.5
O1^i^—Ni1—O7	90.70 (7)	C2—C3—H3	119.5
O4^i^—Ni1—O7	89.35 (7)	C3—C4—C5	119.2 (3)
O4—Ni1—O7	90.65 (7)	C3—C4—H4	120.4
O1—Ni1—O7^i^	90.70 (7)	C5—C4—H4	120.4
O1^i^—Ni1—O7^i^	89.30 (7)	C6—C5—C4	121.0 (2)
O4^i^—Ni1—O7^i^	90.65 (7)	C6—C5—H5	119.5
O4—Ni1—O7^i^	89.35 (7)	C4—C5—H5	119.5
O7—Ni1—O7^i^	180.0	C5—C6—C7	120.7 (3)
O6^i^—Mn1—O1	177.99 (8)	C5—C6—H6	119.7
O6^i^—Mn1—N1^i^	90.59 (8)	C7—C6—H6	119.7
O1—Mn1—N1^i^	87.90 (7)	O3—C7—C6	117.3 (2)
O6^i^—Mn1—O2	101.75 (7)	O3—C7—C2	124.3 (2)
O1—Mn1—O2	79.66 (7)	C6—C7—C2	118.4 (2)
N1^i^—Mn1—O2	166.99 (7)	O5—C8—N1	120.2 (2)
O6^i^—Mn1—O9	87.57 (8)	O5—C8—C9	120.09 (19)
O1—Mn1—O9	93.84 (8)	N1—C8—C9	119.7 (2)
N1^i^—Mn1—O9	93.75 (8)	C10—C9—C14	118.9 (2)
O2—Mn1—O9	90.88 (8)	C10—C9—C8	117.4 (2)
O6^i^—Mn1—O10	88.50 (8)	C14—C9—C8	123.6 (2)
O1—Mn1—O10	90.20 (8)	C11—C10—C9	121.3 (2)
N1^i^—Mn1—O10	90.55 (8)	C11—C10—H10	119.4
O2—Mn1—O10	85.74 (8)	C9—C10—H10	119.4
O9—Mn1—O10	174.20 (8)	C10—C11—C12	119.7 (2)
O3—Mn2—O4	176.03 (8)	C10—C11—H11	120.2
O3—Mn2—N2	88.18 (8)	C12—C11—H11	120.2
O4—Mn2—N2	93.66 (7)	C13—C12—C11	120.3 (2)
O3—Mn2—O5	98.67 (7)	C13—C12—H12	119.9
O4—Mn2—O5	79.85 (7)	C11—C12—H12	119.9
N2—Mn2—O5	171.17 (7)	C12—C13—C14	121.4 (2)
O3—Mn2—O8	93.89 (8)	C12—C13—H13	119.3
O4—Mn2—O8	89.72 (7)	C14—C13—H13	119.3
N2—Mn2—O8	86.94 (8)	O6—C14—C13	116.8 (2)
O5—Mn2—O8	87.05 (7)	O6—C14—C9	124.7 (2)
O3—Mn2—O11	87.30 (8)	C13—C14—C9	118.5 (2)
O4—Mn2—O11	89.15 (7)	O8—C15—O7	124.9 (2)
N2—Mn2—O11	91.20 (8)	O8—C15—C16	118.4 (2)
O5—Mn2—O11	94.65 (7)	O7—C15—C16	116.7 (2)
O8—Mn2—O11	177.77 (7)	C15—C16—H16A	109.5
N2—O1—Mn1	115.37 (13)	C15—C16—H16B	109.5
N2—O1—Ni1	116.20 (13)	H16A—C16—H16B	109.5
Mn1—O1—Ni1	121.46 (8)	C15—C16—H16C	109.5
C1—O2—Mn1	111.39 (15)	H16A—C16—H16C	109.5
C7—O3—Mn2	126.39 (15)	H16B—C16—H16C	109.5
N1—O4—Mn2	112.52 (12)	O9—C17—H17A	109.5
N1—O4—Ni1	114.00 (12)	O9—C17—H17B	109.5
Mn2—O4—Ni1	109.98 (8)	H17A—C17—H17B	109.5
C8—O5—Mn2	111.06 (13)	O9—C17—H17C	109.5
C14—O6—Mn1^i^	129.29 (16)	H17A—C17—H17C	109.5
C15—O7—Ni1	126.98 (16)	H17B—C17—H17C	109.5
C15—O8—Mn2	132.85 (17)	O10—C18—H18A	109.5
C17—O9—Mn1	127.6 (2)	O10—C18—H18B	109.5
C17—O9—H9O	111 (3)	H18A—C18—H18B	109.5
Mn1—O9—H9O	113 (3)	O10—C18—H18C	109.5
C18—O10—Mn1	126.2 (2)	H18A—C18—H18C	109.5
C18—O10—H10O	111 (3)	H18B—C18—H18C	109.5
Mn1—O10—H10O	108 (3)	O11—C19—H19A	109.5
C19—O11—Mn2	127.54 (17)	O11—C19—H19B	109.5
C19—O11—H11O	104 (3)	H19A—C19—H19B	109.5
Mn2—O11—H11O	128 (3)	O11—C19—H19C	109.5
C20—O12—H12A	109.5	H19A—C19—H19C	109.5
H13A—O13—H13B	97.8	H19B—C19—H19C	109.5
C8—N1—O4	111.81 (18)	O12—C20—H20A	109.5
C8—N1—Mn1^i^	129.84 (16)	O12—C20—H20B	109.5
O4—N1—Mn1^i^	118.35 (13)	H20A—C20—H20B	109.5
C1—N2—O1	111.30 (18)	O12—C20—H20C	109.5
C1—N2—Mn2	131.90 (16)	H20A—C20—H20C	109.5
O1—N2—Mn2	115.94 (14)	H20B—C20—H20C	109.5
O2—C1—N2	120.4 (2)		
			
N1^i^—Mn1—O1—N2	−163.95 (16)	Mn2—O3—C7—C6	153.19 (19)
O2—Mn1—O1—N2	12.24 (15)	Mn2—O3—C7—C2	−30.0 (3)
O9—Mn1—O1—N2	102.43 (16)	C5—C6—C7—O3	178.2 (2)
O10—Mn1—O1—N2	−73.40 (15)	C5—C6—C7—C2	1.2 (4)
N1^i^—Mn1—O1—Ni1	−14.29 (10)	C3—C2—C7—O3	−178.3 (2)
O2—Mn1—O1—Ni1	161.89 (11)	C1—C2—C7—O3	−0.3 (4)
O9—Mn1—O1—Ni1	−107.91 (11)	C3—C2—C7—C6	−1.4 (4)
O10—Mn1—O1—Ni1	76.26 (10)	C1—C2—C7—C6	176.6 (2)
N2—Mn2—O3—C7	32.0 (2)	Mn2—O5—C8—N1	−11.7 (3)
O5—Mn2—O3—C7	−153.54 (19)	Mn2—O5—C8—C9	168.76 (16)
O8—Mn2—O3—C7	118.9 (2)	O4—N1—C8—O5	−4.0 (3)
O11—Mn2—O3—C7	−59.2 (2)	Mn1^i^—N1—C8—O5	175.17 (16)
Mn2—O4—N1—C8	18.3 (2)	O4—N1—C8—C9	175.55 (18)
Ni1—O4—N1—C8	144.44 (16)	Mn1^i^—N1—C8—C9	−5.3 (3)
Mn2—O4—N1—Mn1^i^	−160.92 (9)	O5—C8—C9—C10	−2.0 (3)
Ni1—O4—N1—Mn1^i^	−34.81 (17)	N1—C8—C9—C10	178.5 (2)
Mn1—O1—N2—C1	−14.5 (2)	O5—C8—C9—C14	176.4 (2)
Ni1—O1—N2—C1	−165.81 (15)	N1—C8—C9—C14	−3.1 (3)
Mn1—O1—N2—Mn2	174.82 (9)	C14—C9—C10—C11	0.5 (4)
Ni1—O1—N2—Mn2	23.53 (19)	C8—C9—C10—C11	179.0 (2)
Mn1—O2—C1—N2	2.2 (3)	C9—C10—C11—C12	0.1 (4)
Mn1—O2—C1—C2	−178.51 (18)	C10—C11—C12—C13	−0.5 (5)
O1—N2—C1—O2	7.6 (3)	C11—C12—C13—C14	0.4 (4)
Mn2—N2—C1—O2	176.28 (17)	Mn1^i^—O6—C14—C13	−167.37 (18)
O1—N2—C1—C2	−171.72 (19)	Mn1^i^—O6—C14—C9	14.8 (4)
Mn2—N2—C1—C2	−3.0 (3)	C12—C13—C14—O6	−177.8 (2)
O2—C1—C2—C3	14.8 (4)	C12—C13—C14—C9	0.2 (4)
N2—C1—C2—C3	−165.9 (2)	C10—C9—C14—O6	177.2 (2)
O2—C1—C2—C7	−163.2 (2)	C8—C9—C14—O6	−1.2 (4)
N2—C1—C2—C7	16.1 (3)	C10—C9—C14—C13	−0.6 (3)
C7—C2—C3—C4	0.8 (4)	C8—C9—C14—C13	−179.1 (2)
C1—C2—C3—C4	−177.3 (2)	Mn2—O8—C15—O7	2.6 (4)
C2—C3—C4—C5	0.2 (4)	Mn2—O8—C15—C16	−176.5 (2)
C3—C4—C5—C6	−0.5 (4)	Ni1—O7—C15—O8	13.4 (4)
C4—C5—C6—C7	−0.2 (4)	Ni1—O7—C15—C16	−167.5 (2)

Symmetry code: (i) −*x*+1, −*y*+1, −*z*+1.

### Hydrogen-bond geometry (Å, º)

**Table:**

*D*—H···*A*	*D*—H	H···*A*	*D*···*A*	*D*—H···*A*
O9—H9*O*···O12^ii^	0.82 (2)	1.82 (2)	2.630 (4)	172 (4)
O10—H10*O*···O7^i^	0.84 (2)	1.97 (3)	2.713 (3)	148 (4)
O11—H11*O*···O5^iii^	0.83 (2)	1.95 (2)	2.778 (2)	178 (4)
O12—H12*A*···O8	0.84	1.94	2.743 (3)	159

Symmetry codes: (i) −*x*+1, −*y*+1, −*z*+1; (ii) −*x*+2, −*y*+1, −*z*+1; (iii) −*x*+1, −*y*+2, −*z*+1.
